# The impact of increased nuchal translucency on pregnancy outcomes: A retrospective cohort study in Qatar

**DOI:** 10.5339/qmj.2025.103

**Published:** 2025-12-04

**Authors:** A. Adnan, V. Chilaka, R. Musa, J. Vazhiyilethil, A. Ibrahim, N. Mohammad Ali, SA. Qureshi, SB. Ahmed, N. Khenyab, F. Minisha, T. Farrell

**Affiliations:** 1 Department of Obstetrics and Gynaecology, Women’s Wellness and Research Centre, Hamad Medical Corporation, Doha, Qatar; 2 Department of Women’s Imaging, Women’s Wellness and Research Centre, Hamad Medical Corporation, Doha, Qatar; 3 College of Medicine, Qatar University, Qatar *Email: aadnan@hamad.qa

**Keywords:** Nuchal translucency, congenital anomalies, chromosomal anomalies, pregnancy outcomes, neonatal health, Qatar

## Abstract

**Background:**

Nuchal translucency (NT) measurement by ultrasound is used in the first trimester as a screening tool for genetic, chromosomal, and structural anomalies. As the NT measurement increases, the risk of an underlying abnormality also rises. This study aims to evaluate the significance of increased NT measurements within a local cohort, examining their associations with adverse pregnancy outcomes and their potential role in guiding clinical interventions.

**Methods:**

Pregnancies with first-trimester fetal NT measurements greater than 2.5 mm were included. Participants were categorized into five groups based on NT measurements: ≤3.4 mm, 3.5–4.4 mm, 4.5–5.4 mm, 5.5–6.4 mm and ≥6.5 mm. The outcomes evaluated included chromosomal anomalies confirmed by invasive testing (such as Trisomy 21), major congenital anomalies involving any major organ system, and miscarriage or termination of pregnancy before 24 weeks of gestation. Gestational age at delivery, birthweight, small-for-dates (SFD) status, and admission to the neonatal intensive care unit (NICU) were evaluated for infants born after 24 weeks of gestation without congenital anomalies.

**Results:**

The median NT measurement among the 290 women in the study was 3.7 mm, ranging from 2.9 mm (25th centile) to 7.4 mm (75th centile). Overall, 43.5% of the participants were in the lowest NT category, while 15.9% were in the highest category. Maternal age, body mass index, and nationality were comparable between the groups. The odds of chromosomal anomalies increased with higher NT measurements, with odds ratio (OR) ranging from 2.50 to 4.02 (*p* < 0.05), compared to the lowest NT group. Similarly, the odds of major congenital anomalies (OR: 2.20–4.20; *p* < 0.05), multiple anomalies (OR: 5.61–8.19 in the highest three categories; *p* < 0.005), and miscarriages (OR: 5.04–14.9 in the highest three categories; *p* < 0.001) all increased with rising NT measurements. The odds of most chromosomal anomalies increased with NT, except for Trisomy 21, which was similar across the groups. Participants in the two lowest NT groups had 12.7- and 7.5-fold higher odds (both *p* < 0.001) of achieving a pregnancy beyond 24 weeks without anomalies compared to those in the highest NT group. Among pregnancies resulting in viable non-anomalous deliveries, there were no significant differences in the gestational age at delivery, birthweight, incidence of SFD, or NICU admission.

**Conclusion:**

Adverse outcomes, including chromosomal and congenital anomalies, increased with higher NT measurements, with a significant difference observed beyond 3.4 mm. These findings highlight the importance of early NT screening and targeted interventions to improve perinatal outcomes.

## 1. INTRODUCTION

Nuchal translucency (NT) refers to the sonographic appearance of a subcutaneous fluid collection at the back of the fetal neck, between the skin and the underlying soft tissue. It becomes visible and measurable on ultrasound between 11 and 14 weeks of gestation.^1,2^ NT can be measured either alone or as part of a combined prenatal aneuploidy screening, which integrates NT measurements with maternal age and serum markers such as free β-human chorionic gonadotropin (β-hCG) and pregnancy-associated plasma protein A (PAPP-A).^2^ The ideal timing for NT measurement is between 11+0 and 14+0 weeks of gestation, corresponding to a crown–rump length (CRL) of 45–84 mm.^3^ The thickness of the NT is related to the CRL and is considered increased when the measurement exceeds the 95th percentile for a given CRL. An NT value between 2.5 and 3 mm is generally regarded as within the normal range for this marker.^4^ Studies have shown that an NT measurement above the 99th percentile or exceeding 3.5 mm is associated with an increased risk of adverse pregnancy outcomes.^5,6^ Such adverse outcomes include major cardiac defects, structural anomalies, genetic syndromes, poor fetal growth, miscarriage, and intrauterine fetal demise.^5,7^ These risks have also been shown to increase in proportion to the size of the NT.^1,7,8^

The exact reason for the increased NT in these abnormalities remains poorly understood. During fetal development, various hemodynamic changes and the maturation of the lymphatic system significantly influence the degree of nuchal fluid accumulation. NT may become thickened due to abnormal lymphatic development, extracellular matrix disorders, and structural malformations, which can ultimately result in cardiac failure.^7,8^

It is well recognized that the ranges of various parameters can differ significantly across populations due to genetic, ethnic, and environmental factors. Global standards may therefore provide NT reference ranges that lack the precision to accurately predict the prevalence of anomalies in different populations.

Understanding population-specific NT values is crucial for accurate prenatal screening and the early detection of potential pregnancy complications.

This study aims to evaluate the significance of increased NT measurements in a local cohort, examining their associations with adverse pregnancy outcomes and their potential to guide clinical interventions, particularly in cases with mildly elevated NT.

## 2. METHODOLOGY

### 2.1. Study design

This retrospective cohort study was conducted at the Women’s Wellness and Research Center (WWRC) in Qatar to examine the relationship between increased NT values and their impact on pregnancy outcomes. The study used data from pregnant women attending WWRC, where routine first-trimester prenatal screenings included NT measurements. The center serves a diverse population reflecting Qatar’s demographic composition, including both local and expatriate populations.

### 2.2 Study population

The study used data from 290 pregnant women referred to the Fetal Medicine Unit (FMU) at WWRC due to increased NT detected during the first-trimester scans between January 1, 2015, and November 29, 2022.

**2.2.1. Inclusion criteria**

Inclusion criteria included pregnancies with clearly documented NT and a viable fetus at the time of ultrasound.

**2.2.2. Exclusion criteria**

Exclusion criteria included pregnancies with incomplete outcome data, non-viable fetuses, or cases presenting with significant confounding conditions, such as fetal hydrops, as these factors could significantly skew the results.

### 2.3. Ethical considerations

The study received approval (MRC-01-22-808) from the Institutional Review Board (IRB) of Hamad Medical Corporation.

### 2.4. Data collection

Anonymized data were retrospectively collected from the FMU at WWRC by reviewing the charts of women referred for increased NT measurements (>2.5 mm) between January 1, 2015, and November 29, 2022. The study data included key demographic variables, including maternal age, body mass index (BMI), nationality, and parity. The medical history focused on previous pregnancies affected by congenital anomalies and on pre-existing medical conditions, such as diabetes. First-trimester ultrasound data included CRL, NT measurements, and any associated findings indicative of structural abnormalities or potential genetic syndromes. The study exposure—NT measurements—were categorized into five groups: 2.5–3.4, 3.5–4.4, 4.5–5.4, 5.5–6.4, and ≥6.5 mm. The study outcome measures—documented pregnancy outcomes—included chromosomal anomalies, congenital anomalies, and miscarriages or terminations. Additionally, data on prenatal testing (both non-invasive and invasive) were recorded.

### 2.5. Statistical analysis

Statistical analyses were performed using “Stata statistical software, Release 18.” Continuous variables were summarized using means and standard deviations or medians with interquartile ranges (IQRs), while categorical variables were presented as frequencies and percentages. Logistic regression models were used to estimate odds ratios (ORs) with corresponding 95% confidence intervals (CIs) for outcomes across NT categories. Statistical significance was set at *p* < 0.05.

## 3. Results

### 3.1. Demographics

The study included 290 pregnant women, and their baseline characteristics are presented in Table 1. The mean maternal age was 32.6 ± 5.8 years (range: 19–47 years). Approximately two-thirds of the participants were multiparous (*n* = 167, 57.8%), and 34.5% (*n* = 100) were Qatari nationals. The mean maternal booking BMI was 28.8 ± 6.2 kg/m^2^, with 35.4% (*n* = 102) classified as obese (BMI ≥ 30 kg/m^2^). A history of previous pregnancies with congenital anomalies was reported in 15.9% (*n* = 46), and 19.0% of the participants had been diagnosed with maternal diabetes.

The mean gestational age at the time of NT measurement was 13.0 ± 0.8 weeks (range: 9.6–15.7 weeks). The median NT measurement was 3.7 mm (IQR: 2.9–5.4 mm, range: 2.5–36 mm). Overall, 43.5% (*n* = 126) of cases had NT values in the 2.5–3.4 mm range, 22.4% (*n* = 65) in the 3.5–4.4 mm range, 10.0% (*n* = 29) in the 4.5–5.4 mm range, 8.3% (*n* = 24) in the 5.5–6.4 mm range, and 15.9% (*n* = 46) had measurements ≥6.5 mm. The mean CRL at NT measurement was 68.0 ± 10.3 mm (range: 41–98 mm).

Suspicion of congenital anomalies was noted in 44.1% (*n* = 128) of pregnancies, with 25.5% (*n* = 74) presenting a single major anomaly and 18.6% (*n* = 54) presenting multiple anomalies. Non-invasive prenatal testing (NIPT) was performed in 37.6% (*n* = 109) of cases, yielding high-risk results in 8.6% (*n* = 25). Invasive testing was conducted in 37.9% (*n* = 110) of pregnancies via chorionic villus sampling (CVS) and in 18.3% (*n* = 53) via amniocentesis.

### 3.2. Relationship between NT and CRL

Table 2 provides NT measurements in the cohort according to CRL, highlighting the dynamic relationship between fetal growth (CRL) and NT thickness during the first trimester.

The median NT values remain relatively stable across most CRL categories, ranging between 3.4 mm and 4.6 mm, suggesting that NT measurements do not increase linearly with fetal size. The highest median NT value (4.6 mm) is observed in the smallest CRL category (40–44 mm).

### 3.3. NT measurements and outcomes

Analysis of pregnancy outcomes across the five NT measurement categories (Table 3) reveals a clear trend: as NT thickness increases, the proportion of normal pregnancies decreases. In the overall cohort of 290 pregnancies, 109 (37.6%) progressed beyond 24 weeks without any congenital anomalies. In the baseline NT group (2.5–3.4 mm), 54.8% of pregnancies reached this milestone, compared to only 8.7% in the ≥6.5 mm category. Concurrently, the rate of miscarriages or terminations rose from 17.7% in the baseline group to 76.2% in the highest NT category. Similar patterns were observed for chromosomal anomalies, major congenital anomalies, and multiple anomalies.

Analysis of the associations showed that higher NT measurements were significantly associated with increased odds of adverse outcomes, including chromosomal anomalies, congenital anomalies, and pregnancy loss. The strength of these associations increased with NT thickness, with the ≥6.5 mm category exhibiting the highest risk for most outcomes. For chromosomal anomalies considered as a combined category, the odds were 2.5 times higher for NT measurements of 3.5–4.4 mm (OR: 2.50, 95% CI: 1.22–5.13, *p* = 0.012), increasing to 3.44 times for 4.5–5.4 mm (OR: 3.44, 95% CI: 1.41–8.42, *p* = 0.007), and 3.0 times for NT ≥ 6.5 mm (OR: 3.00, 95% CI: 1.38–6.54, *p* = 0.006) compared to the baseline category. Subcategories, including Trisomy 21 and other chromosomal anomalies, were observed but did not consistently reach statistical significance in individual comparisons.

A similar trend was observed for major congenital anomalies, with odds approximately two times higher for NT measurements of 3.5–4.4 mm (OR: 2.22, 95% CI: 1.10–4.50, *p* = 0.026), increasing to 4.20 times for NT ≥ 6.5 mm (OR: 4.20, 95% CI: 1.99–8.85, *p* < 0.001) compared to the 2.5–3.4 mm reference category. Multiple congenital anomalies showed a strong association with NT, with odds eight times higher in the 4.5–5.4 mm group (OR: 8.19, 95% CI: 3.07–21.85, *p* < 0.001) and five times higher in the ≥6.5 mm group (OR: 5.61, 95% CI: 2.30–13.71, *p* < 0.001) compared to the reference category.

Pregnancy loss was also significantly associated with higher NT measurements, with odds increasing from five times higher for NT 4.5–5.4 mm (OR: 5.04, 95% CI: 2.00–12.66, *p* = 0.001) to nearly 15 times higher for NT ≥ 6.5 mm (OR: 14.88, 95% CI: 6.3–35.1, *p* < 0.001) compared to the baseline category. Conversely, the odds of pregnancies progressing beyond 24 weeks without congenital anomalies decreased significantly with higher NT measurements, with odds 12.7 times higher in the 2.5–3.4 mm group compared to the ≥6.5 mm group (OR: 12.7, 95% CI: 4.30–37.58, *p* < 0.001).

### 3.4. Proportion of congenital anomalies across NT categories

**Figure 1:**
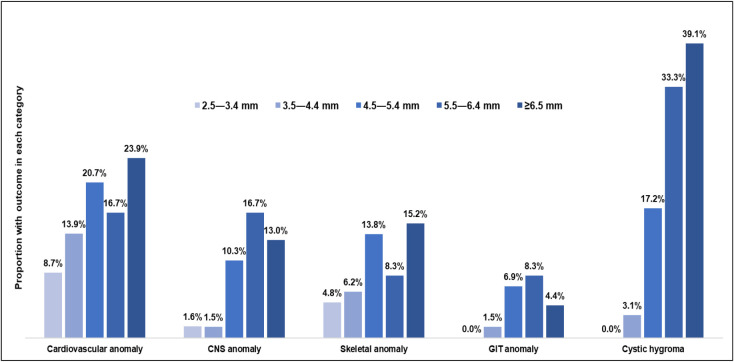
Proportion of women with congenital outcomes in each NT measurement category. Cardiovascular system; CNS, Central nervous system; GIT, Gastrointestinal.

We examined the association between NT categories and the prevalence of various congenital anomalies (Figure 1). Increased NT thickness is associated with a higher prevalence of anomalies, particularly cardiovascular anomalies, which reach 23.9% in the ≥6.5 mm group. Skeletal anomalies also show an upward trend, increasing from 4.8% in the 2.5–3.4 mm category to 15.2% in the ≥6.5 mm category. Similarly, gastrointestinal (GIT) anomalies are more common in higher NT categories, peaking at 8.3% in the 5.5–6.4 mm group. Cystic hygroma is strongly associated with increased NT measurements, rising sharply to 33.3% in the 5.5–6.4 mm category and 39.1% in the ≥6.5 mm group. Conversely, central nervous system (CNS) anomalies are rare in the lower NT categories (2.5–3.4 mm and 3.5–4.4 mm) but show higher prevalence in the 5.5–6.4 mm and ≥6.5 mm categories, at 16.7% and 13.0%, respectively. Overall, the findings indicate that higher NT measurements are strongly associated with congenital anomalies—particularly cystic hygroma, skeletal anomaly, and cardiovascular system anomaly—highlighting the important role of NT as a screening tool in early pregnancy in Qatar.

### 3.5. Proportion of pregnancy outcomes across NT categories

**Figure 2:**
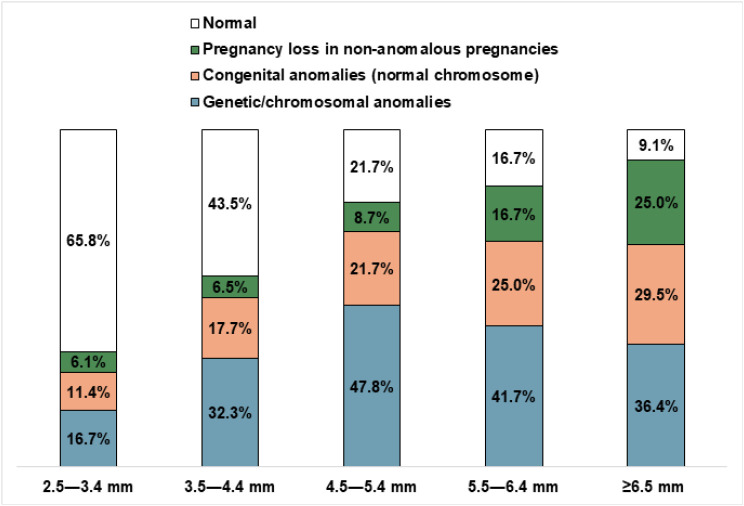
Proportion of women with pregnancy outcomes in each NT category. Total numbers *N* = 290, 2.5–3.4 mm = 126 (missing = 12), 3.2–4.4 mm = 65 (missing = 3), 4.5–5.4 mm = 29 (missing = 6), 55–6.4 mm, ≥6.5 mm = 46 (missing = 2).

Figure 2 illustrates the distribution of pregnancy outcomes across different NT categories. The proportion of normal pregnancy outcome decreases with increasing NT, from 65.8% in the 2.5–3.4 mm group to 9.1% in the ≥6.5 mm group. Conversely, pregnancy loss in non-anomalous pregnancies increases from 6.1% in the 2.5–3.4 mm to 25.0% in the ≥6.5 mm category. The incidence of congenital anomalies with normal chromosomes steadily increased from 11.4% in the lowest NT group to 29.5% in the highest NT category. Genetic/chromosomal anomalies rise sharply from 16.7% in the 2.5–3.4 mm group, peaking at 47.8% in the 4.5–5.4 mm category, and then slightly declining to 36.4% in the ≥6.5 mm group.

## 4. DISCUSSION

This study examines the relationship between NT measurements and pregnancy outcomes in a diverse population in Qatar, providing a comprehensive evaluation of increased NT and its clinical implications. To our knowledge, this is the first study conducted in Qatar to explore population-specific NT measurements and their association with adverse pregnancy outcomes. The findings are particularly significant in the context of the region’s unique demographic characteristics, including a high rate of consanguinity, which may influence the prevalence of chromosomal and structural anomalies. By highlighting these associations, this study provides valuable insights that highlight the need for tailored prenatal screening strategies to improve maternal and fetal health outcomes.

The findings of this study hold significant clinical importance, as they reinforce the use of NT as a key screening tool for the early detection of chromosomal and structural anomalies. The retrospective cohort design of this study enabled a systematic analysis of data from 290 pregnancies managed at the WWRC, focusing on NT measurements obtained between 9.6 and 15.7 weeks of gestation. However, an increased NT measurement beyond 14 + weeks of gestation may still be acceptable. The study population, reflective of Qatar’s unique demographic composition, included local and expatriate women, providing insights into a population not previously explored in similar studies. Although the Qatari population was not studied exclusively, the findings remain highly relevant to the clinical context of Qatar. Demographic and geographic factors—such as widespread access to prenatal services across primary and tertiary care settings, environmental conditions like extreme heat, and population-level factors including high rates of consanguinity—may all influence pregnancy outcomes.

A significant association was demonstrated between increased NT measurements and adverse pregnancy outcomes. Higher NT values were associated with increased risks of chromosomal and structural anomalies, as well as pregnancy loss, with the highest risks observed in the ≥6.5 mm category. These findings align with previous research, which has consistently reported that NT values exceeding standard thresholds are predictive of genetic syndromes, such as Trisomy 21, and structural defects, particularly cardiovascular anomalies.^6,9^ In our cohort, chromosomal anomalies were detected in 15.1% of cases with NT measurements of 2.5–3.4 mm, increasing to 30.8% for NT 3.5–4.4 mm and 34.8% for NT ≥ 6.5 mm. These results closely align with the detection rates in earlier studies, which reported that chromosomal abnormalities occur in approximately 20–30% of cases with NT ≥ 3.0 mm, rising to 40% for NT ≥ 4.5 mm.^10,11^ The chromosomal abnormalities identified in our study—Trisomy 21, Trisomy 18, and Trisomy 13—are consistent with those reported in the literature.^12^ Studies by Bellai-Dussault et al.,^13^ Zhou et al.,^12^ and Lugthart et al.,^14^ have similarly highlighted the proportional relationship between NT thickness and the risk of chromosomal anomalies. Regarding structural anomalies, this study observed major anomalies in 25.5% of cases with increased NT, aligning with prior studies that report a similar prevalence of congenital anomalies. Senat et al. documented structural anomalies in 27% of patients with increased NT,^15^ while Boutot et al. reported anomalies in 28.7%, with cardiac and urogenital anomalies being the most common.^16^ This highlights the persistent association between increased NT thickness and the prevalence of structural anomalies observed over the past two decades.

Pregnancy loss was observed in 32.41% of cases in our study. Variations in reported pregnancy loss rates, ranging from 7.14%^17^ to 58.3%,^18^ are likely influenced by factors such as the presence of structural anomalies, chromosomal abnormalities, differences in NT thresholds, and variations in diagnostic protocols.^19^ Notably, studies have reported higher pregnancy loss rates when increased NT is accompanied by structural anomalies, emphasizing the need for individualized counseling and management.

In this study, cystic hygroma was strongly associated with increased NT, particularly in the ≥6.5 mm group. This finding is consistent with the existing literature, highlighting cystic hygroma as a marker for chromosomal and structural anomalies.^20^

Although increased NT is associated with a higher risk of chromosomal abnormalities, structural malformations, and adverse outcomes, it is important to note that not all cases lead to a poor prognosis. Approximately 50–70% of fetuses with isolated increased NT (≥3.5 mm) and a normal karyotype are reported to have uneventful pregnancies and deliver healthy neonates.^21^ Therefore, increased NT should not be considered a definitive diagnosis, but rather an indication for thorough follow-up. Current international guidelines recommend targeted fetal assessment, including a detailed anomaly scan at 18–22 weeks of gestation and fetal echocardiography at 20–22 weeks.^22^ Genetic evaluation is also recommended, either through invasive testing (e.g., CVS or amniocentesis) or NIPT, depending on the clinical context.^23^ These steps help in the early diagnosis or exclusion of major malformations and genetic syndromes, enabling informed counseling and appropriate perinatal management.^18^

This study highlights the strong association between increased NT measurements and adverse pregnancy outcomes, reaffirming NT as a key parameter in early prenatal screening in Qatar.

## 5. CLINICAL IMPLICATIONS

This study highlights the importance of NT measurements as a key screening tool in prenatal care. The strong association between increased NT and adverse pregnancy outcomes highlights the need for routine NT screening, supported by genetic testing and advanced imaging, including fetal echocardiography. Such measures enable early identification of high-risk pregnancies, facilitating timely counseling and appropriate interventions. In Qatar, NT screening is part of routine prenatal care and is offered free of charge across the public healthcare system, including both primary and tertiary care settings. This ensures high accessibility and cost-effectiveness, making it a feasible and equitable screening strategy for all pregnant women within the national healthcare framework. Tailored prenatal care protocols, especially in populations with unique demographic characteristics such as high rates of consanguinity, can further improve maternal and fetal outcomes.

## 6. FUTURE RECOMMENDATIONS

Future research should explore the genetic and molecular mechanisms underlying the association between increased NT and adverse outcomes, using advanced diagnostic tools such as chromosomal microarray analysis and whole-exome sequencing. Longitudinal studies are needed to evaluate the long-term developmental outcomes of pregnancies with increased NT in the absence of chromosomal or structural anomalies. Additionally, establishing population-specific NT reference ranges and integrating them into national screening programs could enhance diagnostic accuracy and policy effectiveness. Collaborations between policymakers and healthcare providers are essential to develop cost-effective, standardized prenatal care strategies tailored to regional demographics.

## 7. STRENGTHS AND LIMITATIONS

This study has several notable strengths, including the use of a robust dataset and detailed stratification of NT categories, allowing for a comprehensive analysis of the relationship between increased NT measurements and adverse pregnancy outcomes. It provides valuable population-specific data, addressing a critical gap in the literature by providing insights into the diverse population of Qatar.

However, certain limitations should be considered. The retrospective design may introduce selection bias, and missing data in a small subset of cases could impact the generalizability of the findings. Additionally, the smaller sample sizes in certain NT categories limited the ability to perform adjusted analyses, which may have affected the precision of the reported associations. Future prospective studies with larger cohorts are needed to validate these findings and provide deeper insights into the implications of increased NT; however, conducting such a study in Qatar may be restricted by the relatively small sample size available.

## 8. CONCLUSION

This study highlights the critical role of NT as a key screening tool for identifying adverse pregnancy outcomes. Increased NT measurements are strongly associated with chromosomal anomalies, structural defects, and pregnancy loss, emphasizing the need for routine screening and timely interventions to improve maternal and fetal outcomes. Future research should investigate the underlying mechanisms and refine screening practices tailored to local populations.

## LIST OF ABBREVIATIONS

**Table:** 

β-hCG	β-Human Chorionic Gonadotropin
BMI	Body Mass Index
CRL	Crown–Rump Length
CVS	Chorionic Villus Sampling
FMU	Fetal Medicine Unit
NIPT	Non-Invasive Prenatal Testing
NT	Nuchal Translucency
PAPP-A	Pregnancy-Associated Plasma Protein A
SFD	Small for Dates

## ACKNOWLEDGMENTS

We would like to thank all the clinicians and sonographers involved in the care of the patients included in this study. Special thanks are extended to the Fetal Medicine Units and Department of Women’s Imaging at the Women’s Wellness and Research Centre, Hamad Medical Corporation, Doha, Qatar.

## COMPETING INTEREST

The authors have no conflicts of interest to declare.

## Figures and Tables

**Table 1.** Cohort characteristics.

Cohort characteristics		Total *N* = 290
Maternal age in years (mean ± SD)		32.6 ± 5.8 Range = 19–47
Maternal parity (*n*, %*N*)	Nulliparous (0)	56 (19.4%)
	Multiparous (1–3)	167 (57.8%)
	Grand multiparous (≥4)	66 (22.8%)
Qatari nationality (*n*, %*N*)		100 (34.5%)
Maternal booking body mass index in kg/m^2^ (mean ± SD)		28.8 ± 6.2 Range = 16.5–1.2
Maternal obesity at booking (BMI ≥ 30 kg/m^2^) (*n*, %*N*)		102 (35.4%)
Any previous pregnancy with a congenital anomaly (*n*, %*N*)		46 (15.9%)
Maternal diabetes		55 (19.0%)
Gestational age at NT measurement in weeks (mean ± SD)		13.0 ± 0.8 Range = 9.6–15.7
NT in mm (median, IQR)		3.7 (2.9–5.4) Range = 2.5–36
NT categories (*n*, %*N*)	2.5–3.4 mm	126 (43.5%)
	3.5–4.4 mm	65 (22.4%)
	4.5–5.4 mm	29 (10.0%)
	5.5–6.4 mm	24 (8.3%)
	≥6.5 mm	46 (15.9%)
CRL at the time of NT, in mm (mean ± SD)		68.0 ± 10.3 Range = 41–98
Suspicion of congenital anomalies	None	162 (55.9%)
	One major anomaly	74 (25.5%)
	Multiple anomalies	54 (18.6%)
NIPT done		109 (37.6%)
High-risk NIPT result		25 (8.6%)
Invasive testing done	CVS	110 (37.9%)
	Amniocentesis	53 (18.3%)

SD: Standard deviation; Parity: Previous viable pregnancies; NT: Nuchal translucency; Range: Smallest value to largest value; Interquartile range (IQR): 25–75%; CRL: Crown–rump length; NIPT: Non-invasive prenatal testing; CVS: Chorionic villus sampling.

**Table 2.** NT measurements in the cohort according to CRL.

CRL measurements (mm)	Gestational age in weeks (Median)	NT measurements (mm)		
		5th centile	Median	95th centile
40–44	11.9	4.0	4.6	5.1
45–49	11.6	2.7	3.7	7.0
50–54	12.3	2.9	4.0	15.0
55–59	12.4	2.6	4.0	12.0
60–64	12.6	2.5	4.2	11.0
65–69	13.0	2.5	4.0	10.0
70–74	13.3	2.5	3.5	7.0
75–79	13.6	2.5	3.4	11.7
80–84	13.9	2.7	3.4	6.6
85–89	13.8	2.5	3.4	16.0
90–94	14.1	3.1	3.9	6.1
95–100	13.9	2.5	3.9	7.0

CRL: Crown–rump length; NT: Nuchal translucency.

**Table 3.** Pregnancy outcomes (proportions and ORs with 95% confidence intervals) in the NT groups.

Outcomes	Parameters	2.5–3.4 mm *N* = 126	3.5–4.4 mm *N* = 65	4.5–5.4 mm *N* = 29	5.5–6.4 mm *N* = 24	≥6.5 mm *N* = 46
Chromosomal anomalies	*n* (%*N*)	19 (15.1%)	20 (30.8%)	11 (37.9%)	10 (41.7%)	16 (34.8%)
	OR (95% CI)	Baseline	**2.50 (1.22–5.13)**	**3.44 (1.41–8.42)**	**4.02 (1.56–10.37)**	**3.00 (1.38–6.54)**
	*p*-value	–	**0.012**	**0.007**	**0.004**	**0.006**
Trisomy 21	*n* (%N)	8 (12.3%)	10 (20.8%)	4 (18.2%)	4 (25.0%)	6 (14.3%)
	OR (95% CI)	Baseline	1.88 (0.68–5.18)	1.58 (0.43–5.88)	2.38 (0.61–9.18)	1.19 (0.38–3.70)
	*p*-value	–	0.225	0.492	0.210	0.767
Other chromosomal anomalies (Trisomy 13, 18, Turners, etc.)	*n* (%N)	11 (8.7%)	10 (15.4%)	7 (24.1%)	6 (25.0%)	10 (21.7%)
	OR (95% CI)	Baseline	1.90 (0.76–4.74)	**3.33 (1.16–9.52)**	**3.48 (1.15–10.6)**	**2.90 (1.14–7.39)**
	*p*-value	–	0.169	**0.025**	**0.028**	**0.025**
Major congenital anomalies	*n* (%N)	21 (16.7%)	20 (30.8%)	12 (41.4%)	9 (37.5%)	21 (45.7%)
	OR (95% CI)	Baseline	**2.22 (1.10–4.50)**	**3.53 (1.47–8.47)**	**3.00 (1.16–7.76)**	**4.20 (1.99–8.85)**
	*p*-value	–	**0.026**	**0.005**	**0.023**	**<0.001**
Multiple congenital anomalies	*n* (%N)	10 (7.9%)	11 (16.9%)	12 (41.4%)	7 (29.2%)	15 (32.6%)
	OR (95% CI)	Baseline	2.36 (0.95–5.90)	8.19 (3.07–21.85)	4.78 (1.60–14.23)	5.61 (2.30–13.71)
	*p*-value	–	0.066	<0.001	0.005	<0.001
Miscarriages/termination of pregnancy before viability (missing = 25)	*n* (%N)	20 (17.7%)	14 (22.6%)	13 (52.0%)	15 (69.6%)	32 (76.2%)
	OR (95% CI)	Baseline	1.36 (0.63–2.92)	**5.04 (2.00–12.66)**	**10.63 (3.87–29.2)**	**14.88 (6.3–35.1)**
	*p*-value	–	0.436	**0.001**	**<0.001**	**<0.001**
Pregnancies above 24 weeks, without congenital anomaly *N* = 109	*n* (%N)	69 (54.8%)	27 (41.5%)	5 (17.2%)	4 (16.7%)	4 (8.7%)
	OR (95% CI)	**12.7 (4.30–37.58)**	**7.46 (2.39–23.3)**	2.19 (0.54–8.93)	2.1 (0.48–9.27)	**Baseline**
	*p*-value	**<0.001**	**0.001**	0.276	0.327	–

NT: Nuchal translucency; OR: Odds ratio; CI: Confidence intervals; *p* < 0.05 Considered significant; Unadjusted logistic regression models. The bold values in the table represents the significant values, *p* < 0.05.
